# Roles of Myosin-Mediated Membrane Trafficking in TGF-β Signaling

**DOI:** 10.3390/ijms20163913

**Published:** 2019-08-12

**Authors:** Chih-Ling Chung, Shun-Ban Tai, Tsung-Hui Hu, Jih-Jung Chen, Chun-Lin Chen

**Affiliations:** 1 Department of Biological Sciences, National Sun Yat-sen University, Kaohsiung 80424, Taiwan; 2 Division of Rheumatology, Immunology and Allergy, Department of Internal Medicine, Zuoying Branch of Kaohsiung Armed Forces General Hospital, Kaohsiung 81342, Taiwan; 3 Department of Marine Biotechnology and Resources, National Sun Yat-sen University, Kaohsiung 80424, Taiwan; 4 Division of Hepato-Gastroenterology, Department of Internal Medicine, Kaohsiung Chang Gung Memorial Hospital, Kaohsiung 83301, Taiwan; 5 Faculty of Pharmacy, School of Pharmaceutical Sciences, National Yang-Ming University, Taipei 11221, Taiwan; 6 Department of Medical Research, China Medical University Hospital, China Medical University, Taichung 40402, Taiwan

**Keywords:** unconventional myosin, TGF-β, endocytosis, subcellular trafficking, lipid-rafts, clathrin-coated pits

## Abstract

Recent findings have revealed the role of membrane traffic in the signaling of transforming growth factor-β (TGF-β). These findings originate from the pivotal function of TGF-β in development, cell proliferation, tumor metastasis, and many other processes essential in malignancy. Actin and unconventional myosin have crucial roles in subcellular trafficking of receptors; research has also revealed a growing number of unconventional myosins that have crucial roles in TGF-β signaling. Unconventional myosins modulate the spatial organization of endocytic trafficking and tether membranes or transport them along the actin cytoskeletons. Current models do not fully explain how membrane traffic forms a bridge between TGF-β and the downstream effectors that produce its functional responsiveness, such as cell migration. In this review, we present a brief overview of the current knowledge of the TGF-β signaling pathway and the molecular components that comprise the core pathway as follows: ligands, receptors, and Smad mediators. Second, we highlight key role(s) of myosin motor-mediated protein trafficking and membrane domain segregation in the modulation of the TGF-β signaling pathway. Finally, we review future challenges and provide future prospects in this field.

## 1. Introduction

Through protein kinase receptors and Smad mediators, transforming growth factor-β (TGF-β) is involved in a wide range of biological processes such as embryonic development, morphogenesis, immune regulation, cell differentiation, wound healing, and inflammation. Furthermore, impairment in the regulation of the TGF-β signaling pathway may cause a broad range of illnesses, such as cardiovascular disease, tissue fibrosis, cancer, and congenital diseases. Three mammalian isoforms of TGF-β, namely TGF-β1, β2, and β3, are encoded by different genes, but they function through the same signaling system [1]. All levels of the TGF-β signaling pathway are optimized for modulating TGF-β family signal transduction. TGF-βs are secreted in an inactive form [2]. Thus, TGF-β1, β2, and β3 are synthesized as precursors comprising a propeptide (also named latency associated peptide (LAP)) and the mature domain. Subsequently, the LAP is removed by the convertase family of endoproteases, producing a mature homodimer protein. The LAP stays associated with the remaining domain and forms a latent complex. The latent complex forms a multiprotein complex with integrins and extracellular matrix (ECM) proteins and retains TGF-βs in the inactive form. The proteolytic processing controls the bioavailability of active TGF-βs by releasing TGF-β into intercellular spaces. Dimerized TGF-β exerts its functions by binding to type II receptor (TβRII) serine/threonine kinases, followed by subsequent recruitment of type I receptors (TβRI) on the cell surface. After phosphorylation by TβRII, TβRI subsequently propagates the signal through phosphorylation of Smad 2/3 proteins. Phosphorylation allows Smad 2/3 proteins to form heteromeric complexes and allows Smad 4 proteins to be translocated into the nucleus. By associating with transcription factors, Smad complexes regulate the expression of target genes [3,4]. Although the canonical Smad-dependent pathway mediates TGF-β signaling, TGF-β signaling is also initiated by Smad-independent signaling pathways, such as Erk, JNK, and p38 MAPK kinase pathways [5]. Together, Smad and non-Smad signaling pathways modulate cellular functions. In pathological processes, such as tumor progression [6], TGF-β is a cytokine that is known for its “double-edge sword” role in carcinogenesis; that is, it has both tumor suppressor and oncogenic activities [7]. In normal epithelial cells and in the early stage of carcinogenesis, TGF-β acts as a potent inducer of growth inhibition. The events of TGF-β-induced growth arrest are marked by the induction of the expression of CDK inhibitors p15INK4B and p21CIP1, which prevent cell cycle progression [8,9]. Once the tumor cell has undergone certain genetic and/or epigenetic changes that attenuate the growth suppressive pathway of TGF-β, targeted overexpression of TGF-β1 can provide tumorigenic advantages, such as driving malignant progression and metastasis [10]. TGF-β promotes cell proliferation, which induces angiogenesis and inhibits immune responses in a tumor microenvironment in the late stage of tumor development. As an immunosuppressive cytokine, TGF-β inhibits the development, proliferation, and activation of immune cells. Targets of TGF-β include T cells (CD4+ effector T cells and CD8+ cytotoxic T cells), NK cells, and macrophages [11,12,13,14]. In addition to its inhibitory effects on T cells, TGF-β promotes the generation of regulatory T cells that inhibit effector T cells, which eventually regulate the activation of NK cells and macrophages [15].TGF-β suppresses both innate and adaptive immune systems and creates an immunotolerant microenvironment, which is advantageous for tumor development. Additionally, TGF-β enhances the ability of cells to migrate and invade. This is achieved through epithelial to mesenchymal transition (EMT), in which epithelial cells change from cuboidal to an elongated spindle and invasive phenotype. During EMT, epithelial cells lose their E-cadherin and ZO-1 protein localization on the plasma membrane, and the expression of vimentin, fibronectin, and N-cadherin are upregulated, which increases cell mobility. Although EMT is an essential event in embryonic development and is induced by TGF-β, it is also closely related to pathological contexts in adults. TGF-β induces EMT in several cell types, including breast epithelial cells [16], squamous carcinoma cells [17], ovarian adenosarcoma cells [18], and melanoma cells [19,20]. The effects are cell-autonomous, as TGF-β is secreted by the tumor cell [21]. Transfection of dominant-negative TβRII into highly metastatic mesenchymal mouse colon carcinoma cells attenuated TGF-β-induced EMT [22]. The results indicate that targeting TGF-β signaling is a promising cancer therapy. In addition to the effects on tumor development, TGF-β-induced EMT contributes to organ fibrosis, such as pulmonary fibrosis [23] and hepatic fibrosis [24]. Therapies against TGF-β signaling transduction are potential strategies for TGF-β-related diseases in humans.

Post-translational modification of TGF-β receptors and Smads, such as phosphorylation, ubiquitination, sumoylation, and neddylation, regulate their availability and stability [25,26]. TGF-β signaling induces the expression of I-Smads or Smurf ubiquitinases which induce receptor ubiquitination and further for degradation, thus, establish a negative feedback loop for regulation of TGF-β signaling pathway. Smad7 binds to E3 ubiquitin ligases via interactions with their WW domains and recruits them to activated TβRI. E3 ligase-induced TβRI ubiquitination can direct the receptors into degradation route either via the lysosomal pathway or via the proteasomal pathway owing to the fact that degradation of ubiquitinated receptors is sensitive to both proteasome inhibitors and lysosome inhibitors [26]. Clathrin- or lipid-rafts/caveolae-mediated receptor internalization and recycling is another level of regulation for the TGF-β signaling pathway, which can regulate signaling and protein degradation in the proteasome. However, the roles of receptor endocytosis and intracellular trafficking in TGF-β signaling must be further defined.

## 2. Distribution of TGF-β Receptors in the Plasma Membrane

Evidence suggests that the distribution of receptors in the plasma membrane determines the intracellular routing of receptors and resulting cellular responses [27,28,29]. Similarly, TGF-β receptors were found partitioned in caveolin-1 positive lipid-rafts and non-raft clathrin-coated pits (CCPs), which may determine the different signaling properties of TGF-β receptors. Receptors localized between two domains are dynamically exchangeable, which is regulated through changes in the chemical composition of the plasma membrane, expression of companion proteins, post-translational modifications of the receptors, or extracellular stimuli [27,28,29]. For instance, IL-6 stimulation resulted in increased translocation of TGF-β receptors to the non-lipid-raft fraction and enhanced TGF-β Smad signaling in HK-2 cells [30], and treatment of the cells with hyaluronic acid moved TGF-β receptors into the caveolin-1 positive lipid-raft domain [31]. TβRII binds to ADAM12 (A disintegrin and metalloproteinase domain-containing protein 12) and promotes its internalization via non-raft CCP via a mechanism distinct from its proteolytic activity [32]. A known ubiquitin E3 ligase, c-Cbl (Casitas B-lineage Lymphoma), augments TGF-β signaling by covenant integrating NEDD8 to TβRII at Lys556 and Lys567 [33]. Neddylation of TβRII promotes its endocytosis through CCP while preventing its caveolae-mediated endocytosis [33].

Caveolae are flask-shaped plasma membrane invaginations marked by the presence of caveolin-1 [34]. TβRI directly binds to the scaffolding domain of caveolin-1 [35], recruiting TβRII bind to the caveolin-1 positive membrane rafts [36]. Upon ligand stimulation, TβRI binds to caveolin-1 and suppresses TGF-β-mediated phosphorylation of Smad2, possibly by inhibition of TβRI kinase activity [35]. The protein that interacts with C kinase 1 (PICK1) acts as a scaffold protein that enhances the interaction between TβRI and caveolin-1, which enhances lipid-raft/caveolae localization and further induces TβRI degradation [37]. The GPI-anchored protein CD109 negatively regulates TGF-β signaling by promoting the localization of TGF-β receptors into the caveolar domain in the presence of a ligand [38]. Addition of cholesterol or its triterpenoid analogs switches the localization of TGF-β receptors from non-raft to lipid-raft microdomains in the plasma membrane [39]. A high concentration of cholesterol in the medium inhibits TGF-β responsiveness in cultured cells, including epithelial and endothelial cells, through the recruitment of cell surface TGF-β–TGF-β receptor complexes in lipid-rafts/caveolae of the plasma membrane and promotion of the degradation of these complexes, thus diminishing TGF-β-stimulated signaling and the related cellular responses [39]. Because triterpenoids have a similar chemical structure with cholesterol and have been reported to be inserted into the plasma membrane, our previous studies have demonstrated that triterpenoids modulate TGF-β responsiveness by reorganizing microdomains in the plasma membrane [40,41,42]. All the effects of triterpenoids on TGF-β responsiveness that we studied are rapid and coordinate with triterpenoid-induced TGF-β receptor translocations between lipid-rafts/caveolae and non-lipid raft microdomains in the plasma membrane. The effects start from 0.5 h after treatment of cells with triterpenoids and reach the maximum level at 2 h.

## 3. TGF-β Signaling Is Modulated by Receptor Trafficking

Internalization of TGF-β–TGF-β receptor complexes terminates TGF-β signaling. Accumulated evidence, however, suggests that that endocytosis plays an important role in signal propagation and amplification [43]. Lipid-raft/caveolae-mediated and clathrin-mediated endocytosis (CME) are two primary endocytic pathways that mediate TGF-β receptor internalization on the cell surface [44,45]. Studies have reported that the TGF-β/Smad signaling pathway is initiated in CCPs present in the plasma membrane or early endosomes [44]. In addition to CCPs, low-density and detergent-insoluble membrane microdomains are known as caveolae where the scaffold protein caveolin is associated. Although lipid-rafts/caveolae are utilized as a signaling platform for many signaling pathways, previous studies have reported that lipid-raft/caveolae-mediated endocytosis results in the rapid degradation of TGF-β receptor complexes [44]. For signal propagation, TGF-β receptors need to be expressed on the plasma membrane of cells. However, the receptors predominantly reside in the cytoplasm, and only a minor fraction of TGF-β receptors is expressed on the cell surface and is available for TGF-β binding [27,46,47]. The intracellular pool of TβRI and TβRII receptors may serve as a repository that facilitates the redistribution of receptors to lipid-raft and non-raft CCP on the cell surface; the compartmentalization of TGF-β receptors may diversify signal networks by bringing them into contact with specific interaction partners or substrates and modulating steady TGF-β responsiveness [27].

Improper intracellular trafficking of the TGF-β receptor has been described based on clinical observation and has been reported in connection with several human diseases. Mislocalization has been suggested to contribute to diminishing cell surface TβRII in mitogen-activated CD4+ T cells in patients with Sézary syndrome [48]. Patients exhibit little to no TβRII on the cell surface, and the intracellular pool of the receptors in these cells appears normal [48]. The Shanghai Breast Cancer Study suggested that the expression of p-Smad2 and TβRII in the cytoplasm is predominantly correlated with an invasive histological type and with poor prognosis in breast cancer patients [49]. As mentioned earlier in the text, neddylation of TβRII by c-Cbl promotes clathrin-mediated receptor endocytosis. c-Cbl with a neddylation-defective mutant was found in leukemia patients, which indicates a causal link between aberrant TβRII neddylation and trafficking and leukemia development [32]. TβRII with the E221V/N238I mutant found in human oral squamous cell carcinoma showed impaired endocytosis and enhanced TGF-β signaling [50].

Recently, we reported that among the inhibitors of nonconventional myosins, namely pentachloropseudilin (PClP) [51] and pentabromopseudilin (PBrP) [52], PClP is a reversible and allosteric inhibitor of Myo1c, which inhibits the delivery of TβRII to the plasma membrane through the lipid-raft recycling machinery, resulting in the accumulation of receptors in late endosomes and recycling compartments, and it eventually is rerouted for subsequent degradation in lysosomes. PBrP, a marine antibiotic which was initially purified from the marine bacteria *Pseudomonas bromoutiliz* and *Alteromonas luteoviolaceus*. PBrP selectively inhibits the motor activity of MyoVa. MyoVa has been strongly linked to various stages of exocytosis, namely the capturing, tethering, and transport of secretory vesicles approaching the plasma membrane through the actin-cytoskeleton [52]. MyoVa depletion and PBrP treatment leads to TβRII degradation primarily in lipid-raft membrane fractions and coincided with the decreased TGF-β-induced expression of p Smad 2/3, fibronectin, PAI-1 (plasminogen activator inhibitor-1), and EMT proteins.

## 4. Myosin

Myosins comprise a family of molecular motors that bind to actin filaments to generate force and movement. Myosins have a wide range of functions within the cell, including cell shape regulation, cell motility, organelle trafficking, cell signaling, aiding in endo- and exocytotic processes, membrane domain reorganization, and other eukaryotic motility processes. Myosins have three well-defined regions: the head, neck, and tail domains. The motor (or head) domain can bind to actin filaments, hydrolyze ATP, and generate force. The neck domain consists of varying numbers of IQ motifs, each of which provides a binding site for calmodulin and serves as a lever arm that permits motor domain movements [53]. The tail domain presents variations between different classes of myosins and has various lengths and functions, which depend on the motifs included in its sequence. These motifs may include formation of bipolar filaments; pleckstrin homology (PH) domains for binding to membranes; coiled-coil dimerization regions; and MyTH4-FERM, and Src-homology 3 domain (SH3) domains for protein–protein interactions. Recent phylogenetic analysis of myosins in the human genome grouped these genes into 35 myosin classes [54]. Myosins have been studied and characterized extensively, and much is known about their function in different cellular compartments. However, information on these motor proteins in the regulation of TGF-β signaling remains scarce. In the remaining sections of this review, we summarize the current understanding of myosins in TGF-β signaling, with emphasis on the emerging roles of these molecular motors in receptor intracellular trafficking and membrane domain compartmentalization.

## 5. Myosin I

**Figure 1:**
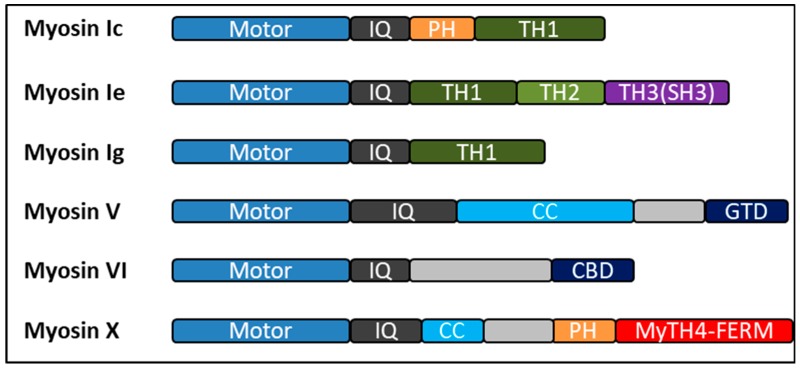
Bar diagrams showing the overall architectures of human myosins discussed in this review. All the myosins have conserved motor heads (blue), different numbers of IQ motifs (gray) followed by a distinct tails with various functional domains. Colored boxes represent different regions predicted by sequence homology: PH, pleckstrin homology domain; TH1, TH2, and TH3, tail homology region 1, 2, and 3; CC, coiled-coil domains; GTD, globular tail domain; CBD, cargo binding domain; SH3, Src-homology 3 domain. Myosin-Va, and myosin-X exist as constitutive dimers and myosin-VI may exist in both monomeric and dimeric forms.

Myosin I is a class of single-headed motor proteins distinct from conventional myosin II, in that myosin I does not form filaments. The single heavy chain is divided into three regions: head, neck, and tail domain (Figure 1). The motor domain is followed by the neck domain. The neck domain is an alpha-helix containing one or more stretches of approximately 29 amino acids, which are referred to as “IQ” motifs [55]. Following the neck domain is the C-terminal tail region. The tail domain of class I myosins contains three conserved regions referred to as tail homology regions 1, 2, and 3 (TH1, TH2, and TH3, respectively). TH1 is rich in basic residues and involved in phospholipid and membrane binding [56]. The TH2 domain is rich in proline, glycine, and alanine/glutamine and contains an ATP-insensitive actin-binding site. The TH3 domain is referred to as the SH3, which is common to several proteins involved in membrane trafficking, actin dynamics, and signal transduction [57]. Myosin I containing only the TH1 domain is delegated to “short-tailed” myosins, whereas those containing all three tail homology domains are referred to “long-tailed” or “classical”. Vertebrates express eight myosin I isoforms, including both short-tailed and long-tailed members. Short-tailed class I myosins include Myo1a, Myo1b, Myo1c, Myo1d, Myo1g, and Myo1h; Myo1e and Myo1f are long-tailed myosins that regulate a number of cellular processes, including the regulation of the cytoskeleton, intracellular transport, cell surface local motion, and regulation of membrane-related events, which includes exocytosis, endocytosis, and phagocytosis [53].

### 5.1. Myo1c

Vertebrate Myo1c is widely distributed in many different cell types and localized to cortical regions of the cell. Immunolocalization and subcellular fractionation studies have demonstrated that in addition to the leading edge and perinuclear region of cells, Myo1c is found in the nucleus [58]. The nuclear isoform (nMyo1c) contains additional 16 amino acids at the N-terminal, which direct its localization to the nucleus, where it is found along with actin. Coimmunoprecipitation and pull-down assays further demonstrate that nMyo1c regulates RNA polymerase II through the formation of the first phosphodiester bond during initiation, and the assays support the role of nMyo1c in transcription [59]. In floxed mice harboring podocytes-specific Myo1c deletion, injury-induced fibrosis was attenuated in the kidney, and podocyte function and morphology were preserved. Cell culture and qPCR analysis further demonstrated that the loss of Myo1c in podocytes specifically dampened both TGF-β canonical and non-canonical pathway signaling, which provides direct evidence for involvement of Myo1c in TGF-β signaling [60]. Crucially, chromatin immunoprecipitation and DNA-protein binding assays showed significant increase of nMyo1c binding at the GDF-15 promoter, suggesting that nMyo1c may directly regulate the transcription of the GDF-15 gene. Because GDF-15 is known to be responsive to TGF-β stimulation and is involved in the pathogenesis of tissue fibrosis, these results support the transcriptional regulatory role of Myo1c, where Myo1c-mediated regulation of TGF-β responsive genes is crucial for disease progression in podocytes [60]. In addition to transcriptional regulation by nMyo1c, the contribution from cytoplasmic Myo1c isoform cannot be excluded, particularly because Myo1c is a key regulator of trafficking of lipid-rafts from intracellular compartments to the plasma membrane [61,62]. Membrane targeting of Myo1c involves a putative PH domain present in the short C-terminal tail [63]. Lipid-rafts are enriched in PI(4,5)P2 [64], which bind to the PH-motifs of Myo1c. Thus, Myo1c may be preferentially distributed to lipid-rafts in the plasma membrane. This function is supported by the finding that Myo1c facilitates the exocytosis and delivery of several raft-associated proteins, such as VEGFR2 [53], aquaporin 2 [65], GLUT4 [66], and NEPH1 [67], to the cell surface. Our recent work identified PClP as a reversible and allosteric inhibitor of Myo1c that inhibits the delivery of TβRII to the plasma membrane through the lipid-raft recycling machinery, resulting in the accumulation of receptors in late endosomes and recycling compartments, and it eventually is rerouted for subsequent degradation in lysosomes [51].

### 5.2. Myo1e

Myo1e is the only “long-tailed” type I myosin that is ubiquitously expressed in mammalian cells. The C-terminal tail of Myo1e contains a TH1 region, which consists of a putative PH domain, a proline-rich TH2, and a Src-homology 3 (SH3) domain. In mammalian cells, immunolocalization studies have demonstrated that Myo1e is localized to clathrin and dynamin positive puncta in the plasma membrane [68]. A previous study demonstrated that Myo1e facilitates normal dynamin and clathrin dynamics, recruits actin polymerizing and regulatory factors to CCPs during the late stages of CME, and promotes cargo trafficking from the plasma membrane to early endosomes [69]. Inhibition of actin assembly and depletion of Myo1e caused reduced transferrin endocytosis and a profound delay in its trafficking to early endosomal compartments. In terms of pathological relevance, high Myo1e expression has been identified as part of the gene signature that predicts poor outcome in patients with basal-like breast cancer; additional meta-analysis shows an inverse correlation between Myo1e expression in grade 1 breast cancer and patient survival, suggesting that Myo1e promotes tumorigenesis [70,71]. Moreover, genome-wide association studies have shown that Myo1e single-nucleotide polymorphisms are associated with keloid formation (i.e., a wound healing reaction with excessive scar formation) [68]. Since Myo1e was suggested to be involved in the step of vesicle scission during CME, which is also involved in promotion of TGF-β signaling. Elevated TGF-β signaling and the Myo1e activity are closely associated with similar pathological outcomes, such as tumorigenesis, keloid, and tissue fibrosis. Therefore, it may be speculated that Myo1e may play a role in both TGF-β receptor internalization and the transport of the receptors to endosomal compartment and further affect TGF-β signaling.

### 5.3. Myo1g

Myosin1g (Myo1g) is a monomeric class I myosin with an N-terminal catalytic motor domain, a neck region that contains IQ motifs, and a C-terminal tail that directly associates with PI(3,4)P2 and PI(3,4,5)P3 in membranes through a putative PH domain [72,73]. Myo1g is predominantly expressed in hematopoietic cells and has been shown to be accumulated in the plasma membrane. Myo1g plays a crucial role in the association between the plasma membrane and the actin-cytoskeleton in lymphocytes. It also plays a role in the phagocytosis of opsonized microbeads in macrophages [73] and is involved in cell spreading and cell adhesion in B lymphocytes [74]. Furthermore, the proteomics analysis of cell compartments from human T and B-lymphocyte cell lines revealed the enrichment of Myo1g in endosomes and exosomes [75,76]. Lopez-Ortega and Santos-Argumedo demonstrated that Myo1g is a lipid-raft-associated motor protein that is crucial for the cellular distribution and trafficking of CD44 [72]. Myo1g participates in the recycling of vesicles enriched in caveolin-1 and GPI-anchored proteins. Depletion of Myo1g results in the misplacement of lipid-rafts and CD44 in the plasma membrane, which suggests a role for Myo1g in the exocytosis of lipid-raft membranes and proteins from an intracellular recycling compartment [72]. Previous studies have revealed an association between the extracellular matrix polysaccharide hyaluronic acid (HA) and TGF-β-induced cellular responsiveness [31]; however, the molecular mechanism is not clear. HA promotes the signaling interaction between the HA receptor CD44 and TβRI in metastatic breast tumor cells. A study from the Phillips research group that used renal proximal tubular cells showed that co-localization of CD44 and TGF-β receptors facilitates modulation of both Smad and non-Smad-dependent TGF-β-mediated events by HA [31]. This suggests that the alteration of Myo1g activity may represent an endogenous mechanism to regulate TGF-β cellular function. More specifically, we hypothesize that Myo1g-mediated alteration of TGF-β signaling is the result of redistribution of TGF-β receptors between the lipid-raft-caveolar compartment and the endosomal signaling compartment.

## 6. Myosin V

**Figure 2:**
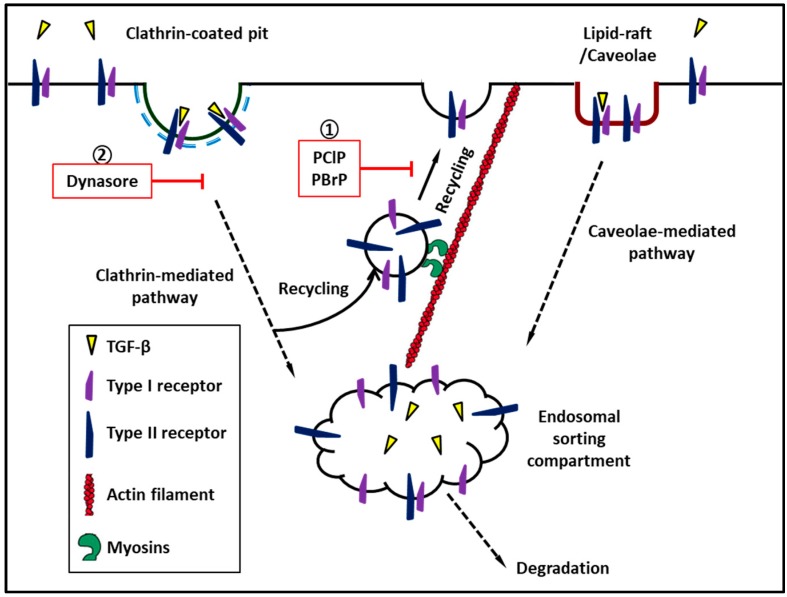
Myosins regulate recycling of transforming growth factor-β receptor to cell surface. ① Pentachloropseudilin and pentabromopseudilin suppress recycle of TGF-β receptor to plasma membrane. ② Dynasore, a cell-permeable inhibitor of dynamin which inhibits clathrin-mediated endocytosis of TGF-β receptors.

Class V Myosin (Myo5) members are two-headed dimeric proteins that contain three types of Myo5, namely Myo5a, Myo5b, and Myo5c. Among these, Myo5a has been studied extensively for its mechanical and enzymatic properties as well as its cellular functions [77,78]. The neck domain is followed by the motor domain and contains six tandem IQ motifs capable of binding multiple light chains. The light chains are always calmodulin or calmodulin-related proteins. The tail region is followed by the neck domain, which comprises of long stretches of a coiled-coil-forming sequence, and Myo5a dimerizes near this stalk region to form a two-headed molecule [53]. The globular tail domain (GTD) is in the distal tail of each heavy chain, which has been implicated in cargo transport [77,78]. Myo5a activity is regulated by molecular folding, in which the GTD folds back and interacts with the motor domains to form a compact molecule [53,78]. Myo5 members are recognized as cargo-carrying, processive motors. It has been recognized that this motor moves progressively along actin filaments through a “head-over-head” lever-arm mechanism that gives 36-nm steps. The function of this protein has been well studied in different cell types and involves the movement of many types of cargo including melanosomes [79], secretory vesicles [80], ER [81], and centrosomes [82]. Myo5 interacts with microfilaments and numerous cytoskeleton components, such as microtubules [83], kinesin, intermediate filaments, and organelle-docking proteins, which are the small G protein complexes. This interaction suggests that Myo5 may be a component of a multiprotein motor complex that provides an “all cytoskeleton drive” for movement of organelles [53]. Our recent study demonstrated that the Myo5 inhibitor PBrP is a potent inhibitor of TGF-β activity. PBrP abrogates TGF-β-stimulated Smad protein phosphorylation and PAI-1 protein expression as well as blocks TGF-β-induced EMT in epithelial cells. PBrP suppresses TGF-β signaling by minimize the cell surface expression of TβRII, and further promotes receptor degradation. Gene silencing approaches suggest that Myo5a plays a crucial role in PBrP-induced TβRII turnover and in the subsequent reduction of TGF-β signaling [52]. Myo5 moves a wide range of receptors intracellularly and regulates their signaling and biological functions, such as glutamate receptor [84], β2-adrenergic receptors [85], hepatocyte growth factor [86], and GLUT4. Thus, the regulatory effects of Myo5 in TGF-β subcellular localization should be analyzed to understand the role of Myo5 in TGF-β signaling (Figure 2).

## 7. Myosin VI

Myosin VI (Myo6) is ubiquitously expressed in mammalian cells and has existed in early life form throughout evolution. Myo6 is the only known motor that moves toward the minus end of actin filaments, and it is involved in a wide range of cellular functions, such as exocytosis, endocytosis, and cytokinesis [87]. Myo6 is believed to transport cargos inward or push actin filaments outward because the plus ends of actin filaments are oriented toward the plasma membrane [87]. Myo6 tail binds to the membranes that contain the second messenger PtdIns(4,5)P2 through the C-terminal cargo binding tail region (CBD) (Figure 1), which resembles the regions identified in other myosin PtdIns(4,5)P2-binding proteins [87]. In HeLa cells, mutation of the PtdIns(4,5)P2 binding site abrogates the targeting of Myo6 and its tail to CCP structures [49]. Many cytoskeletal and endocytic proteins bind to PtdIns(4,5)P2 in the plasma membrane. The protein–lipid interaction regulates the assembly, scission, and uncoating of clathrin-coated vesicles [76,77,78]. PtdIns(4,5)P2 is concentrated at active sites of CME, where it may recruit Myo6, Dab2, and accessory/cytoskeletal proteins to the membrane at the initiation of the CCP assembly. Because Dab2 has been shown to be associated with the type I and type II TGF-β receptors and modulate Smad activation [88], knocking down Dab2 has a substantial impact on TGF-β receptor recycling and subcellular localization. As mentioned before, CME plays a crucial role in the canonical pathway of TGF-β signaling. Thus, it has been speculated that Myo6 has a novel role in TGF-β receptor trafficking and recycling, suggesting that Myo6 is a crucial regulator of TGF-β receptor trafficking between the CCP and the early endosomes.

## 8. Myosin X

Myosin X (Myo10) is an approximately 240-kDa protein that has a structure plan consisting of head, neck, and tail domains. The head domain can bind to actin and hydrolyze ATP to produce force and movement [89]. The neck contains three IQ motifs, each of which is predicted to bind to calmodulin or calmodulin-like light chains. The first segment of the Myo10 tail was initially predicted to form a coiled-coil, suggesting that Myo10 might form dimers [90]. These data suggest a higher possibility of Myo10 undergoing regulated dimerization. Following the coiled-coil region of Myo10 are three PEST regions that are rich in serine, glutamate, proline, and threonine. These sequences are often sites of proteolysis by the calcium-dependent protease calpain [91]. Subsequent to the PEST region of the Myo10 tail is a group of three PH domains. Myo10 is the only known myosin with multiple PH domains in its tail [90]. Using a dot blot assay, a fusion protein consisting of all three PH domains bound to PtdIns(3,5)P2 and PtdIns(3,4,5)P3 with high affinity and with a 10-fold lower affinity to PtdIns(4,5)P2. Following the three PH domains in the tail of Myo10 are the myosin tail homology 4 (MyTH4) domains. MyTH4 domains are relatively short (approximately 150 residues); however, a well-conserved domain was found to bind to microtubules. The ends of the Myo10 tail is the FERM domain, which was originally discovered in Band 4.1, Ezrin, Radixin, and Moesin proteins. Crucially, two-hybrid experiments and pull-down assays showed that the Myo10 FERM domain binds to the NPXY motif in the cytoplasmic domain of β5-integrin, β1-integrin, and β3-integrin. Several classes of unconventional myosins (classes VII, X, XII, and XV) have been reported to share a conserved structural feature in their tail domains—the presence of a MyTH4 domain followed by FERM. MyTH4-FERM myosins have been demonstrated to mediate membrane–cytoskeleton interactions. Recent evidence suggests that Myo10 is involved in filopodia formation, adhesion, phagocytosis, and actin–microtubule interactions [92,93]. Dvornikov et al. conducted phenotypic and transcriptome-wide studies and showed that the stimulation of the lung squamous cell carcinoma (LUSC) cell line SK-MES1 with TGFβ resulted in enhanced migratory feature. By using the next-generation sequencing to analyze the dynamics of gene expression, it was found that TGFβ stimulation coordinates the upregulation of several motility- and actin-cytoskeleton-related genes, including the non-muscle myosins Myo10, Myo1e, and MYH9. Among these the non-muscle myosin, Myo10, exhibits the highest upregulation in a LUSC patient cohort of the Cancer Genome Atlas (TCGA) [92]. Depletion of Myo10 using siRNA abrogated TGF-β-induced collagen gel infiltration of SK-MES1 cells. These observations also support previous findings demonstrating that shRNA knockdown of Myo10 in the MDA-MB-231 cells inhibited Matrigel invasion experiments and in vivo pervasion in lung colonization and mammary fat pads assays [94]. Overall, these results implicate that the biological function of Myo10 may play crucial roles in TGF-β signaling. Myo10 may present as a new molecular target for treating TGF-β-related diseases.

## 9. Conclusions

The multiple functions of the unconventional myosin motors are subjected to regulation in several ways; for example, through PtdIns(4,5)P2 binding, the presence or absence of light chains in both the neck domains, calcium binding, formation of processive dimer, phosphorylation, binding companion proteins, and other signals and modifications, which may together modulate their functions in the cell and further affect the intracellular compartmentalization of TGF-β receptors and TGF-β signaling. Precisely establishing the mechanisms through which myosin functions and regulates the TGF-β signaling pathway in the physio-pathology processes will provide an understanding of how it operates in diseases, such as cancer and tissue fibrosis. This may aid in the development of interventional strategies and the identification of potential drug targets. Since our works demonstrate that myosin inhibitors PClP and PBrP target intracellular trafficking of TGF-β receptors and inhibit TGF-β signaling, these inhibitors could be developed into a broad-spectrum therapeutic agent to treat TGF-β-mediated tissue fibrosis and cancer. Although using myosin inhibitors to cure TGF-β-related diseases is attractive, blocking the key processes such as protein trafficking may eventually prove to be problematic due to intrinsically toxicity to the organism. Moreover, the pleiotropic effects of TGF-β and its role in cell proliferation, tissue homeostasis, and immunity raise concerns regarding potential adverse effects, which must be considered when inducing TGF-β signaling abolishment. Although this approach is still out of therapeutic applications, the values of such inhibitors have been clearly illustrated previously and will continue to be.

## Abbreviations

TGF-β	transforming growth factor-β
ECM	extracellular matrix
Smad	homologies to the Caenorhabditis elegans SMA (“small” worm phenotype) and Drosophila MAD (“Mothers Against Decapentaplegic”)
EMT	epithelial to mesenchymal transition
NEDD	neural precursor cell-expressed, developmentally downregulated
CCPs	clathrin-coated pits
IL-6	interleukin 6
c-Cbl	casitas B-lineage Lymphoma
ADAM12	a disintegrin and metalloproteinase domain-containing protein 12
CME	clathrin-mediated endocytosis
IQ	IQ calmodulin-binding motif
PClP	pentachloropseudilin
PBrP	pentabromopseudilin
PH	pleckstrin homology
SH3	src-homology 3 domain
HA	hyaluronic acid
ZO-1	zonula occludens-1
MAPK	mitogen-activated protein kinase
